# Exercise-Induced Rhabdomyolysis: A Case Report and Literature Review

**DOI:** 10.7759/cureus.10037

**Published:** 2020-08-26

**Authors:** Amira Al Badi, Sara Al Rasbi, Abdullah M Alalawi

**Affiliations:** 1 Medicine, Sultan Qaboos University Hospital, Muscat, OMN

**Keywords:** rhabdomyolysis, exercise, compartment syndrome, creatine kinase, case report

## Abstract

A 19-year-old man presented to the ED with bilateral leg pain and dark discoloration of the urine after he started an intense aerobic exercise. Blood workup showed significantly elevated creatine kinase (CK), acute kidney injury (AKI), and disseminated intravascular coagulation (DIC). The patient had a double-incision, bilateral fasciotomy with debridement to relieve the bilateral, lower-limb, compartment syndrome following admission. Also, his kidney function deteriorated, requiring several sessions of hemodialysis. His hospital stay was complicated by multidrug-resistant (MDR) *Acinetobacter baumannii* bacteremia. After three weeks of hospital admission, the patient was discharged home with a follow-up outpatient physiotherapy for bilateral foot drop, which showed a remarkable recovery eventually. This case highlights the potentially life-threatening risks associated with unaccustomed physical exercise and emphasizing the essential preventive measures to reduce the risk of developing exercise-induced rhabdomyolysis. We present the pathophysiology of exercise-induced rhabdomyolysis, clinical presentation, diagnosis, treatment, and prognosis.

## Introduction

Exertional or exercise-induced rhabdomyolysis is a condition caused by unaccustomed physical exercise and characterized by a breakdown of skeletal muscles that leads to the release of its intracellular components, such as myoglobin and creatine kinase (CK), into the circulatory system [1-2]. It can cause severe complications, including acute kidney injury (AKI), disseminated intravascular coagulation (DIC), compartment syndrome, cardiac arrhythmia, liver dysfunction, and various electrolyte derangements such as hypocalcemia, hypercalcemia, hyperkalemia, hyperphosphatemia, hypomagnesemia, and hyperuricemia, along with mortality risk [3-6]. Exercise-induced rhabdomyolysis occurs in the setting of intense physical exercise, prolonged physical activity or sudden and excessive muscle contractions, and symptoms include change in the color of the urine, muscle ache and pain, headache, and fatigue [1]. Rhabdomyolysis has been increasingly diagnosed worldwide due to the increased popularity of physical activity and exercise [2, 5, 7]. We report a case of a young man who started an intense aerobic exercise before military recruitment, and had exercise-induced rhabdomyolysis, causing severe organ dysfunctions. This report presents the pathophysiology of exercise-induced rhabdomyolysis, clinical presentation, diagnosis, treatment, and prognosis.

## Case presentation

A 19-year-old man, previously healthy and not on any regular medications, presented to the ED at Sultan Qaboos University Hospital (SQUH) in July 2019 with a one-day history of generalized fatigue, bilateral leg pain, and dark discoloration of the urine. There was no history of fever, chills, nausea, vomiting, or diarrhea. Moreover, there was no history of consuming alcohol, anabolic steroid, or drug abuse. His symptoms started a few hours after jogging for around 26 km to improve his fitness before military recruitment. However, he was barely practicing exercise before this attempt. 

On presentation to the ED, he was alert and oriented but appeared to be in pain. He was clinically dehydrated, and his vitals were as follows: temperature 37.0°C, blood pressure 130/70 mmHg, heart rate 100 bpm and regular, and oxygen saturation 100% on room air. He had generalized bilateral lower limb swelling and tenderness, but the neurovascular examination was intact. Chest, cardiovascular, and abdominal examinations were unremarkable.

Laboratory findings are presented in Table 1. As summarized, the patient presented with high CK, AKI (using RIFLE criteria), DIC, and deranged liver enzymes. His initial venous blood gas showed uncompensated high anion gap metabolic acidosis, which could be explained by severe dehydration, AKI, and exhaustion. His initial electrocardiogram (ECG) and chest X-ray were normal. Immediately, using a combination of normal saline and sodium bicarbonate, IV fluid resuscitation was initiated, targeting urine output between 200 and 300 ml/h. Within the first day of admission, he experienced worsening of the bilateral, lower limb swelling and increased pain requiring opioids. Because of the high probability of acute compartment syndrome, the patient underwent double-incision, bilateral fasciotomy with debridement to relieve the bilateral, lower limb, compartment syndrome.

**Table 1:** Blood and urine tests results on the day of admission. aPTT, activated partial thromboplastin time; TT, thrombin time; CK, creatine kinase; WBC, white blood cell; INR, international normalized ratio

Test	Normal range	Admission day
Hemoglobin (g/dL)	11.5-15.5	18.5
Hematocrit (L/L)	0.350-0.450	0.5
WBC (10^9^/L)	2.2-10.0	33.2
Neutrophil count (10^9^/L)	1.0-5.0	30.2
Venous pH	7.35-7.45	7.13
HCO3 (mmol/L)	21.8-26.9	14.7
pCO2 (mmHg)	32.0-45.0	44.4
Anion gap	5.0-13.0	17.3
Lactate (mmol/L)	0.5-1.6	6.5
CK (U/L)	39-308	587,600
Creatinine (µmol/L)	59-104	167
Potassium (mmol/L)	3.5-5.1	4.2
Sodium (mmol/L)	135-145	136
Alanine transaminase (U/L)	0-41	2561
Aspartate aminotransferase (U/L)	0-40	6632
Alkaline phosphatase (mmol/L)	40-129	140
Total bilirubin (µmol/L)	0-17	19
INR	0.91-1.09	2.65
aPTT (second)	26.4-38.1	53
TT (second)	12.8-17.6	24.4
Fibrinogen (g/L)	1.7-3.6	1.4
Calcium (mmol/L)	2.15-2.55	1.74
Uric acid (mmol/L)	0.20-0.45	0.68
Phosphate (mmol/L)	0.81-1.45	2.05
Urine Dipstick:		
Urine glucose		Negative
Urine bilirubin		Negative
Urine blood		Positive (3+)
Urine pH		6
Urine protein		Trace

**Figure 1:**
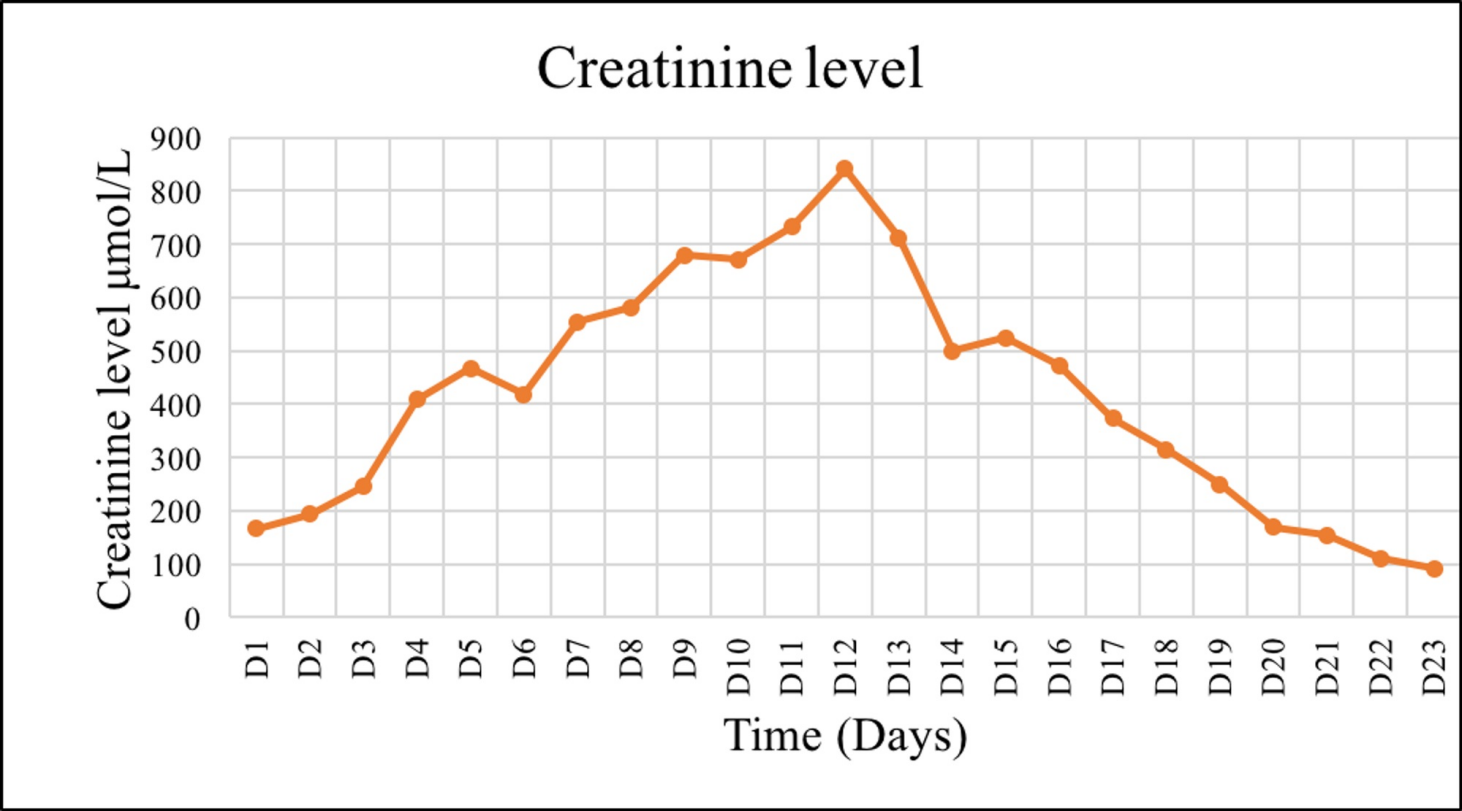
Trend of creatinine level reflecting kidney function during admission.

**Figure 2:**
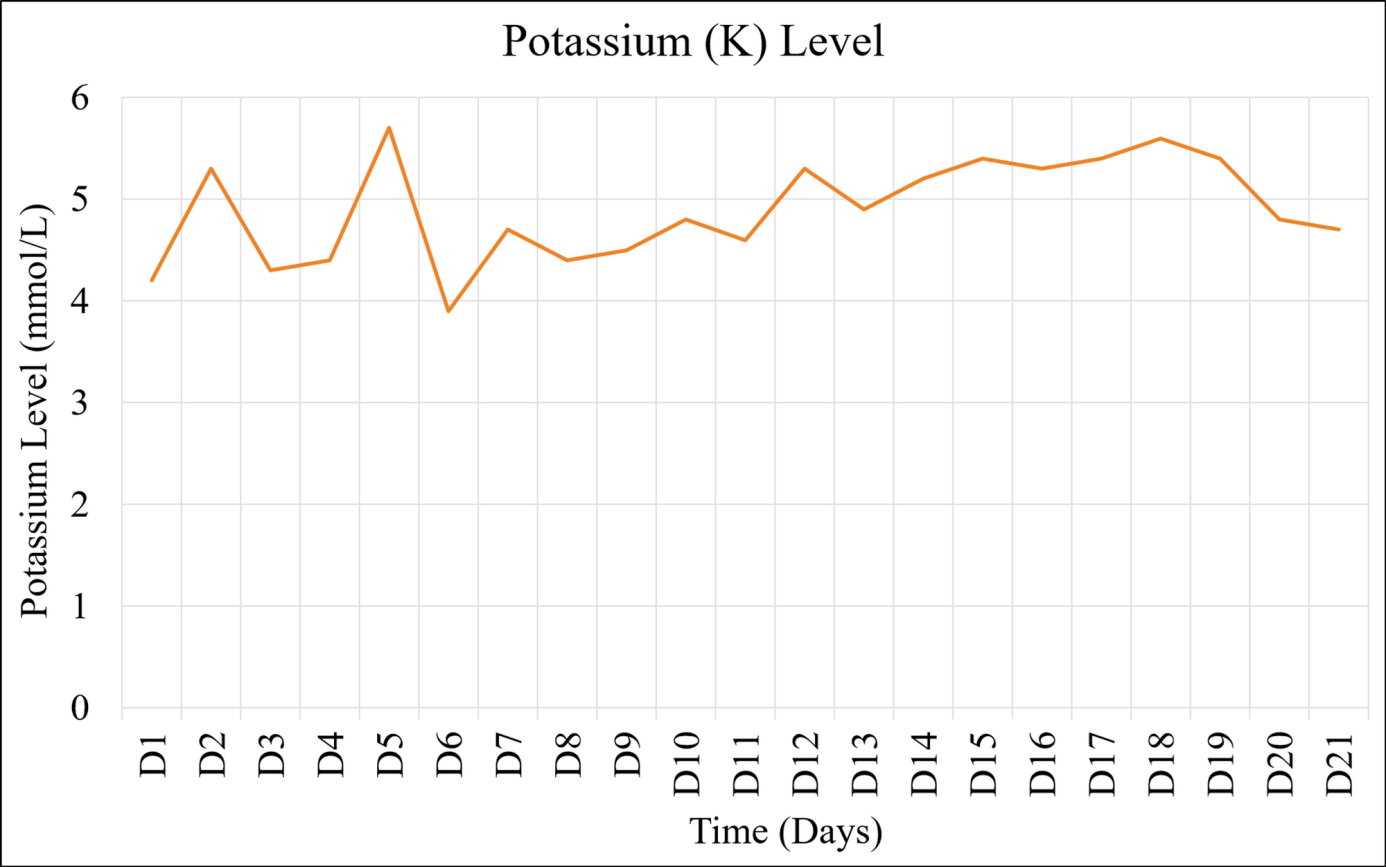
The trend of potassium (K) level during admission.

**Figure 3:**
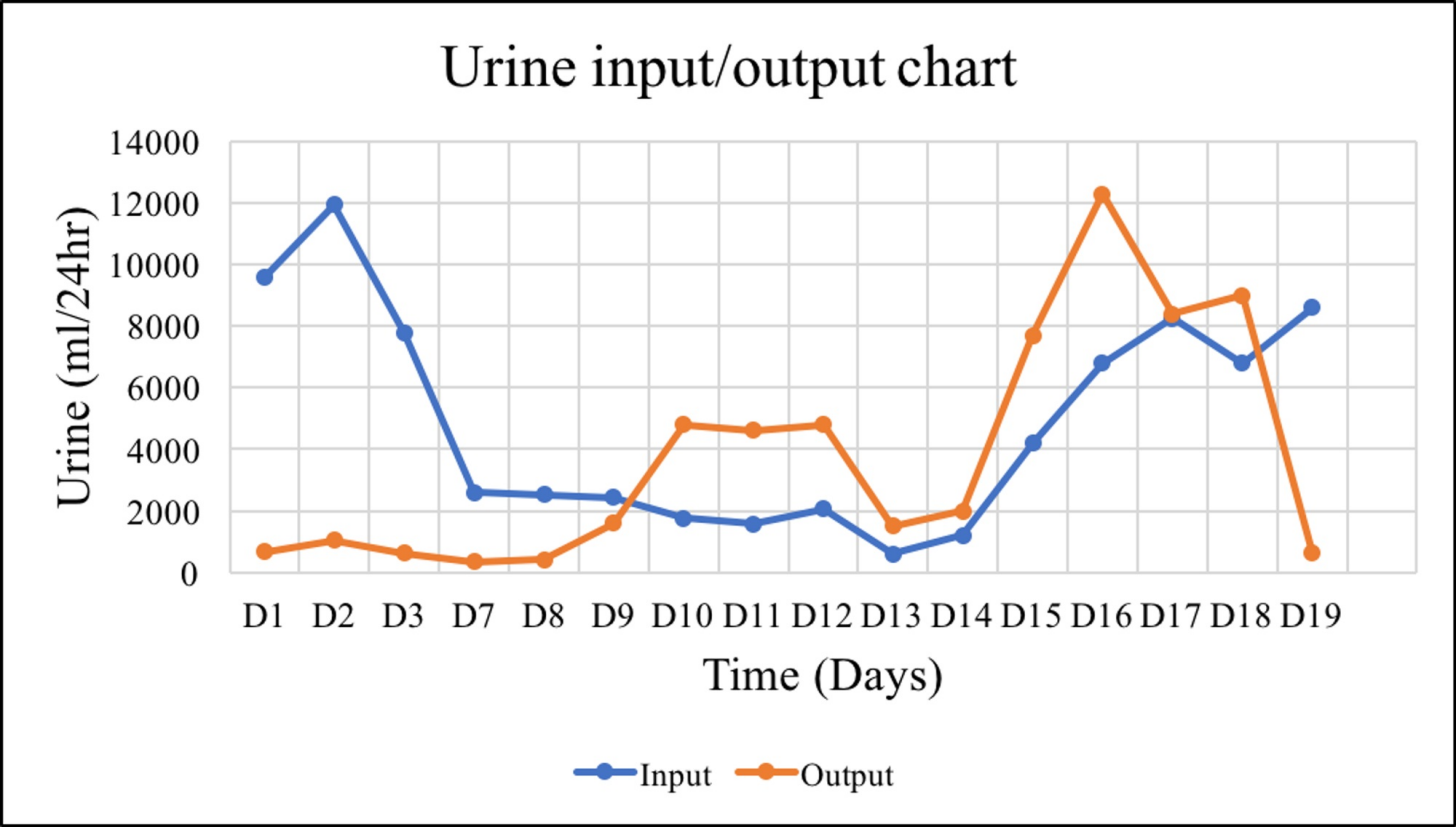
Fluid intake/urine output chart.

Despite adequate rehydration, renal function continued to deteriorate, evidenced by worsening creatinine levels (Figure 1), persistent hyperkalemia (Figure 2), and oliguria ( Figure 3). The patient underwent the first dialysis session within two days after admission. On the ninth day, the patient became unwell, febrile, and tachycardia. After a sepsis workup, the patient was started empirically on meropenem and vancomycin. The blood cultures sampled from the dialysis line and peripheral vein grew for multidrug-resistant (MDR) *Acinetobacter baumannii*. Besides replacing the dialysis line, the patient received a 10-day course of meropenem and a high dose of tigecycline as recommended by the infectious disease team. Overall, the patient showed a clinical and biochemical response to the administered antibiotics.

 

**Figure 4:**
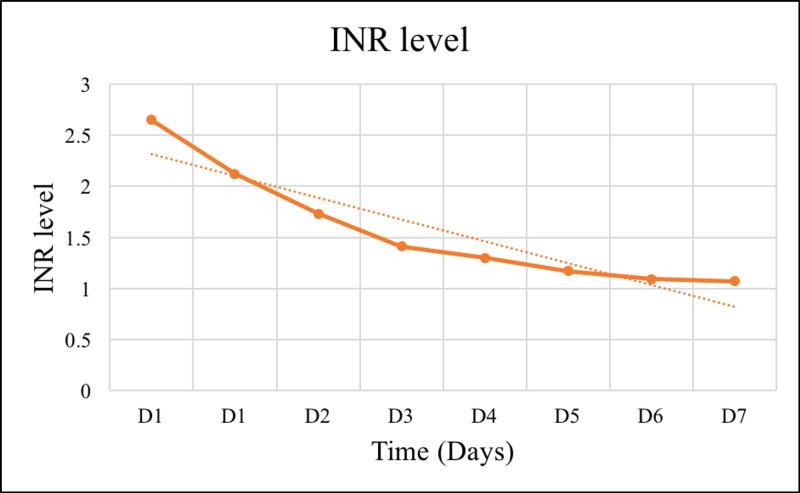
The trend of INR reflects the course and resolution of the coagulopathy. INR, international normalized ratio

**Figure 5:**
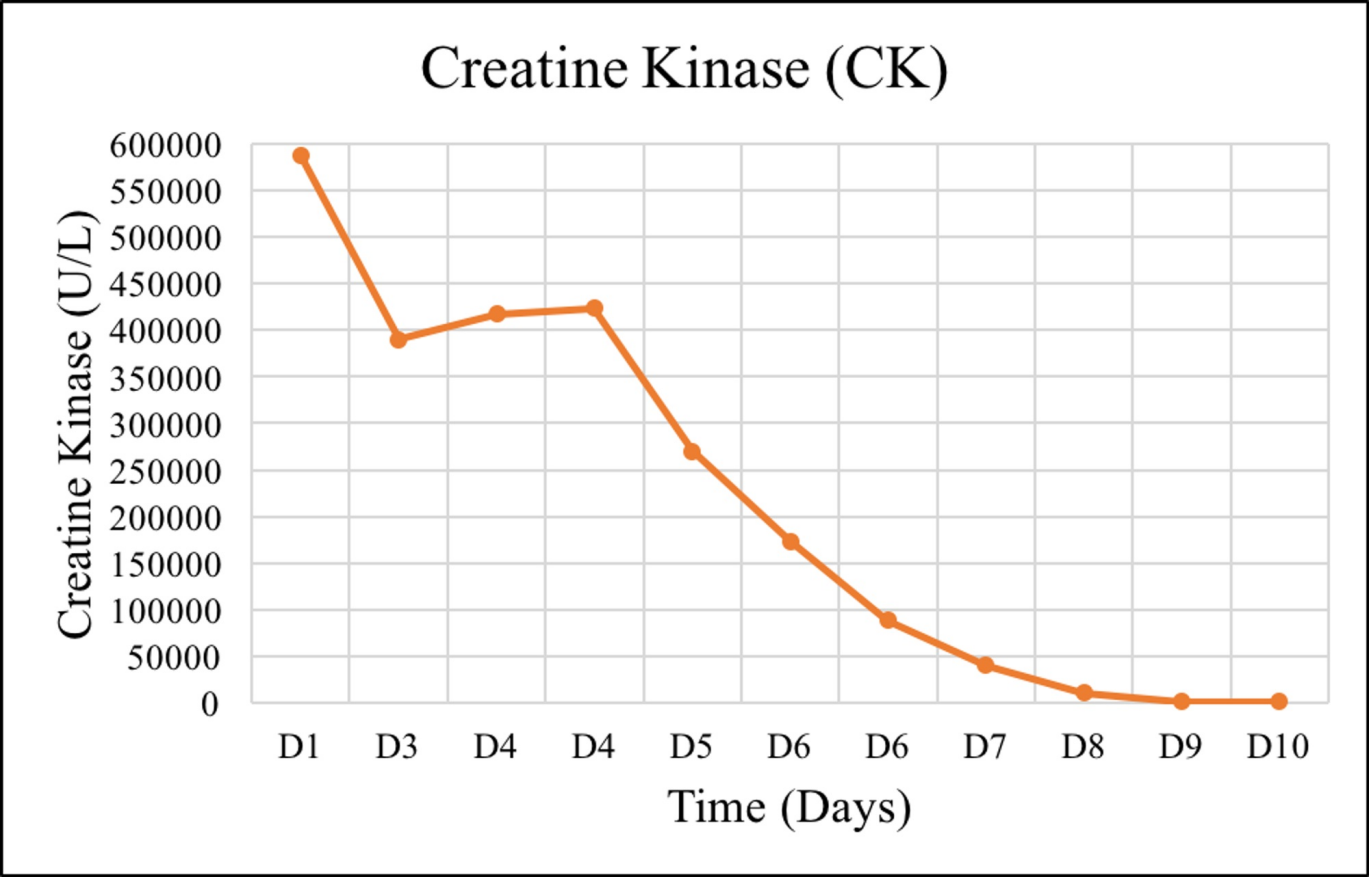
The trend of CK measured during admission. CK, creatine kinase

In summary, the patient completed four sessions of tissue debridement, followed by the closure of fasciotomy wounds and six sessions of hemodialysis. The patient's kidney function (Figures 1-3), coagulopathy measured by INR (Figure 4), and CK (Figure 5) gradually improved. He was discharged as soon as kidney function returned to normal. He also had bilateral foot drop, which improved remarkably with physiotherapy. Otherwise, he remained well upon follow up.

 

## Discussion

Primary factors that cause exercise-induced rhabdomyolysis include poor body fitness combined with prolonged-duration, high-intensity, and weight-bearing exercise (e.g., eccentric contraction, downhill running) [1, 8]. Other predisposing factors include dehydration, hot environment [3], genetic mutations (e.g., Caveolin-3) [4, 9], underlying myopathy [10, 11], drugs such as terbinafine and statin [12], obesity, and tobacco use [13]. Furthermore, sickle cell trait has shown to increase the risk of exercise-induced rhabdomyolysis, even with minor trauma [13].

Intense exercise depletes the adenosine triphosphate (ATP) required by the pumps and channels that regulate calcium (Ca2+) in the sarcoplasmic reticulum, which results in higher resting levels of Ca2+ in muscle cells [1-2]. The increased levels of Ca2+ boost the activity of proteases and phospholipase A2, causing damage to desmin, dystrophin, and other cytoskeleton support structures [1]. The cytoskeleton collapse facilitates muscle membrane tears followed by an increase in the cell membrane permeability, excessive release of intracellular proteins, including CK and myoglobin, an increase in Ca2+ influx, and additional cellular damage [10, 14]. This necrotic process triggers a robust inflammatory response and a subsequent regeneration process [2, 15]. Muscle cellular membrane dysfunction and inflammatory reactions cause an accumulation of intracellular fluids and an increase in muscle compartment pressure, and, thus, worse muscle cell ischemia and local edema eventually lead to acute compartment syndrome [16].

The incidence of AKI in patients with exercise-induced rhabdomyolysis is estimated to be between 10% and 30% [1, 17]. The mortality risk can be as high as 59%, even in an ICU setting [18]. The primary causes of AKI are renal vasoconstriction secondary to myoglobin, hypovolemia, elevated circulating endotoxins and cytokines, and enhanced sympathetic tone and renin-angiotensin-aldosterone system activation [1]. Myoglobin effects contributing to kidney damage include the formation of casts in the distal convoluted tubules and the direct toxicity of myoglobin in the proximal convoluted tubules [3].

Muscle pain, weakness, fatigue, and dark discoloration of urine are classical symptoms of exercise-induced rhabdomyolysis [5]. An initial diagnosis is typically made if CK levels are elevated more than five times the upper limit [5], and there is a history of recent strenuous physical activity. 

Treatment includes an IV infusion of crystalloid fluids using normal saline with or without Ringer's lactate to achieve urine output between 200 and 300 mL/h [5]. If systemic acidosis is present, sodium bicarbonate should also be administered to ensure urine pH is higher than 6.5 to minimize myoglobin-induced kidney damage [3-4]. However, the evidence that suggests urinary alkalization is more effective than hydration alone in reducing AKI incidence [17, 19] is weak. If compartment syndrome is suspected, prompt orthopedic consultation is warranted to evaluate intra-compartmental pressure, and, if necessary, perform a fasciotomy to avoid or minimize damage caused by ischemic necrosis of skeletal muscles [3, 20].

Our patient had the full spectrum of exercise-induced rhabdomyolysis associated complications, accompanied by a hospital-acquired and potentially deadly infection. The patient's exercise routine was unusual, where he jogged more than 26 km per day in hot weather. His body temperature was 37.0°C, which is not consistent with a heatstroke diagnosis. Blood tests also indicated AKI, DIC, and several electrolytes derangements along with deranged liver enzymes. The patient also developed bilateral, lower-limb, compartment syndrome that required a bilateral double fasciotomy and several sessions of wound debridement. Although the adequate and prompt intervention was established, the patient had bilateral foot drop, due to myonecrosis that involved anterior tibial muscles, which improved remarkably with physiotherapy. Despite adequate IV hydration and urinary alkalization, hemodialysis was necessary. His kidney function returned to normal within three weeks after admission. Moreover, during admission, the patient acquired MDR *A. baumannii* line related bacteremia. He was successfully treated with 10 days of IV antibiotics.

## Conclusions

Although the prevalence of exercise-induced rhabdomyolysis has increased, this case has shown potentially added complications such as healthcare-associated infection, which might be life-threatening. Measures such as warming-up and periodic repetition of eccentric exercises, along with sufficient water intake, should be considered for people who exercise for the first time or those with poor body fitness to aim to prevent exertional rhabdomyolysis. 
